# UNDERSTANDING BARRIERS TO PARTICIPATE IN DEMENTIA RESEARCH AMONG OLDER ADULTS IN THE BRONX, NEW YORK

**DOI:** 10.1093/geroni/igae098.3760

**Published:** 2024-12-31

**Authors:** Oshadi Jayakody, Mirnova Ceide, Morgan Grabanski, Claudene George

**Affiliations:** Albert Einstein College of Medicine, Bronx, New York, United States; Albert Einstein College of Medicine, Bronx, New York, United States; Albert Einstein College of Medicine, Bronx, New York, United States; Albert Einstein College of Medicine, Bronx, New York, United States

## Abstract

The Bronx County in New York (NY) has the highest prevalence of dementia in the state and is considered the least healthy of NY counties. This study aims to identify the barriers to participation in dementia research among older adults in the Bronx and explore strategies for improving their research readiness. We are distributing a brief survey to 100 older adults (aged ≥60) visiting ambulatory practice and memory clinics at Montefiore Medical Center and community-based older adult centers in the Bronx. This survey, which will be completed in September 2024, assesses participants’ knowledge of dementia research, awareness of local dementia research studies, willingness to participate in different types of research and barriers for participation. Currently, we have collected survey data from 15 older adults (n=46% White,15% Black, 30% Hispanic). Our preliminary results show that 50% of older adults were aware of available local dementia research studies. Only 17% reported prior participation while 80% reported willingness to participate in future research. Most respondents (81%) were interested in studies investigating cognitive function and healthy aging, whereas 36% were interested in research involving brain imaging, drug trials or lifestyle interventions and genetic studies. The main barrier to participation was limited access to our study centers (55%) followed by lack of time (27%). Interestingly, 82% indicated that free transportation or parking would incentivize their participation. These findings highlight the knowledge gaps and what barriers to address (i.e. enhance accessibility) to improve readiness to participate in future dementia research among older adults in the Bronx.

